# The Role of Quiet Eye Timing and Location in the Basketball Three-Point Shot: A New Research Paradigm

**DOI:** 10.3389/fpsyg.2019.02424

**Published:** 2019-10-30

**Authors:** Joan N. Vickers, Joe Causer, Dan Vanhooren

**Affiliations:** ^1^Faculty of Kinesiology, University of Calgary, Calgary, AB, Canada; ^2^Research Institute for Sport and Exercise Sciences, Liverpool John Moores University, Liverpool, United Kingdom

**Keywords:** vision, motor control, attention, perception-action, expertise, eye tracking, training

## Abstract

We investigated three areas of uncertainty about the role of vision in basketball shooting, the timing of fixations (early, late), the location of fixations (hoop centre, non-centre) and the effect of the defender on performance. We also sought to overcome a limitation of past quiet eye studies that reported only one quiet eye (QE) period prior to a phase of the action. Elite basketball players received the pass and took three-point shots in undefended and defended conditions. Five sequential QE periods were analyzed that were initiated prior to each phase of the shooting action: QE catch, QE arm preparation, QE arm flexion, QE arm extension, and QE ball release. We used a novel design in which the number of hits and misses were held constant by condition, thus leaving the timing and location of QE fixations free to vary across the phases during an equal number of successful and unsuccessful trials. The number of QE fixations accounted for 87% of total fixations. The greatest percent occurred during QE catch (43.6%), followed by QE arm flexion (34.1%), QE arm extension (17.5%) and QE ball release (4.8%). No fixations were found prior to QE arm preparation, due to a saccade made immediately to the target after QE catch. Fixation frequency averaged 2.20 per trial, and 1.25 during the final shooting action, meaning that most participants had time for only one fixation as the shot was taken. Accuracy was enhanced when: (1) an early QE offset occurred prior to the catch, (2) an early saccade was made to the target, (3) a longer QE duration occurred during arm flexion, and (4) QE arm flexion was located on the centre of the hoop, rather than on non-centre locations. Overall, the results provide evidence that vision of the hoop was severely limited during the last phase of the shooting action (QE ball release). The significance of the results is explored in the discussion, along with a QE training program designed to improve three-point shooting. Overall, the results greatly expand the role of the QE in explaining optimal motor performance.

## Introduction

The quiet eye (QE) is defined as the final fixation or tracking gaze that is located on a specific location or object in the task environment within 3° of visual angle (or less) for a minimum of 100 milliseconds (ms). The onset of the QE occurs prior to a critical phase of the movement and the offset occurs when the gaze deviates off the location for a minimum of 100 ms (Vickers, 1996a, b, 2007). Extensive research shows the QE of elite performers is significantly earlier and longer than that of near-elite, or lower skilled performers (Mann et al., 2007; Lebeau et al., 2016; Rienhoff et al., 2016). Since only one final QE period has been reported in most studies to date, critics rightly mention that the majority of fixations may be ignored that play a critical role in performance (Gonzalez et al., 2015). To date, QE studies have defined the QE period relative to a previously identified single critical or final motor phase that has been derived from past QE studies, available biomechanical research, and/or applied technical knowledge. However, it was never intended that only one QE period be considered, but that all QE periods be isolated within a motor task and the one underlying higher levels of performance empirically determined (Vickers, 1992, 2007). We therefore determined five QE periods in the three-point shot, with each having an onset prior to a critical biomechanical phase of the shooting action: QE catch, QE arm preparation, QE arm flexion, QE arm extension, and QE ball release. Our goal was to determine which of these QE periods was most important in contributing to high levels of accuracy in the three-point shot.

Theoretically, the QE is grounded in one of the oldest findings from psychology and neuroscience, which shows there is a delay (later called a latency or reaction time period) that proceeds the initiation of a movement, or phase of the movement (James, 1890/1982; Wundt, 1904; Ladd and Woodworth, 1911). For decades researchers were mystified by the delay, and what may be happening in the brain during this time. For example, Woodworth (1958) commented that “we know–not merely assume–that states of readiness exist in the nerve centers, even though at the present time we cannot do much in the way of describing what goes on in the brain (p. 41)”. With the advent of mobile eye trackers synchronized to external motor cameras in the 1980’s, the fixations of athletes became available for analysis, thus providing insight to what athletes see during the delay period and the effect this has on their performance. Given the complex nature of most motor tasks, multiple sub-phases exist that together combine to carry out the overall task (Schmidt and Lee, 2014). Theoretically, a QE period could exist before each of the sub-phases, with each providing the task information needed to perform effectively and efficiently. Our goal in this paper was to further our understanding of perceptual-motor coordination by empirically isolating which QE period contributed most to high levels of performance in the three-point shot. Our basic hypothesis is that in order for high levels of success to occur in a motor task, a fixation or tracking gaze must be initiated for a long duration on a specific location in the task environment prior to a specific phase of the movement. It is during this time the brain receives the task specific visual information that it needs to organize the extensive neural networks underlying the planning, initiation and on-going control of the movement.

In selecting the three-point shot, we were motivated by the remarkable performance of Stephen Curry, a National Basketball Association (NBA) player who has not only broke previous records in the three-point shot, but also changed how the game of basketball is played. Only rarely does a single athlete emerge who possesses unique abilities that may be physical, visual, or a combination of both. Curry made more three-point shots than any other player during five NBA seasons (2013, 2014, 2015, 2016, and 2017), received two MVP awards (2015 and 2016), and led his team to three NBA championships (2015, 2017, and 2018). He also made more three-point shots in a single season than any other player (402). His three-point accuracy from 2012 to 2019 was 39.9% (NBA, 2019a). Prior to the emergence of Curry, basketball was a big player’s game with the outcome dominated by tall players (NBA average is 6′7″) who took shots from near the basket. The three-point shot is taken further from the basket than any other shot (range 22′–23.9″ in the NBA), thus allowing a relatively small player like Curry (6′3″) to take shots that previously were attempted on few occasions. In 2010 there were only 16 NBA players who made more than 150 three-point shots per season, while in 2018 there were 50 (NBA, 2019c). Clearly the skill needed to shoot from that distance can be acquired, but little is known about the role of vision in the shot. The three-point shot is unique not only because it is taken further from the basket than any other shot in the game, but it is also very fast. From the moment the ball is received until it leaves the finger tips, players at Curry’s level release the ball in 600–800 ms, making it an exceedingly difficult to defend (Waters, 2017). We had elite players with season statistics similar to Curry receive a pass and take three-point shots during an equal number of hits and misses in undefended and defended conditions.

### Timing of Vision in Basketball Shooting

Despite extensive research carried out in basketball shooting (Okazaki et al., 2015; Marques et al., 2018), uncertainty exists about the role of vision in three areas: the timing of vision (early or late), the location of vision (hoop centre, non-centre) and the effect of the defender (undefended, defended). Eye-tracking studies in basketball have resulted in two schools of thought regarding the timing of vision. QE studies report fixations that occur early in the shooting action are most important (Vickers, 1996a, b, 2017; Harle and Vickers, 2001; Wilson et al., 2009; Vine and Wilson, 2011; Klostermann et al., 2017), while ecological, dynamic system studies stress the importance of late “looking” before the ball is released (de Oliveira et al., 2006, 2007, 2008). Theoretically, the results are important, as an early QE supports motor program, open-loop control in which a well learned neural network or motor program is activated and the movement carried out without the use of on-going visual feedback of the target. In contrast, the ecological approach argues that perceived structures in the optic flow field are sufficient to guide motor behavior in an ongoing manner, without reference to internal neural structures or networks. The first QE study was carried out in the basketball free throw and found that elite players fixated the hoop early for an average of 972 ms on hits and 806 ms on misses, while their near-elite teammates averaged 400 ms on hits and 250 ms on misses (Vickers, 1996a, b). Subsequent studies have confirmed these results for high and lower skilled athletes, under conditions of anxiety and in QE training studies where novices are taught the QE characteristics of experts (Vickers, 1996a, b; Harle and Vickers, 2001; Wilson et al., 2009; Rienhoff et al., 2013; Fischer et al., 2015; Klostermann et al., 2017). A number of perceptual/cognitive and/or neural models have been proposed to explain these findings, for example, attention control theory (Eysenck et al., 2007; Wilson et al., 2009; Causer et al., 2011), ventral and dorsal processing (Vickers, 2012; Vickers and Willams, 2017), the inhibition hypothesis (Klostermann et al., 2014; Klostermann, 2019), and EEG/QE/ocular activity (Janelle et al., 2000a, b; Mann et al., 2011; Muraskin et al., 2016; Gallicchio et al., 2018).

The ecological/dynamic systems approach is based on the work of Bernstein (1967) and Gibson (1979) who state that humans perceive action environments directly, unaided by inference, memories, or internal perceptual/cognitive processes. Highly skilled actors, such as elite athletes, directly perceive the affordances in the environment and organize their movements as they move using “the optic flow field, which is the pattern of motion visible at the eye, (which) also informs about motion and immobility, direction of heading, and steering” (de Oliveira, 2016, p. 260). de Oliveira (2016) citing a study by Carlton (1992) also mentions there is a visuomotor delay period (which is the duration it takes for visual information to be used in motor control), but this is due to a physiological delay and not to higher mental processes. The strongest early evidence supporting optic flow came from Lee (1976, 2009) who found that time-to-contact information, or tau (the inverse of the rate of dilation of the object on the retina) was sufficient to guide motor behavior. A number of ecological studies have been carried out in basketball shooting, with one of the first by Oudejans et al. (2002) who identified two styles of shooting, a high style that used information from the basket to the release of the ball, in contrast to a low style similar to that found in QE studies. de Oliveira et al. (2008) in an eye tracking study found that during the low style, the “expert low-style shooters looked comparatively long at the target area when taking free throws, as was the case in previous research” (Vickers, 1996a, b, p. 403). However, when players used a high style they raised the ball above their head and acquired late visual information from the target prior and during ball release. Results showed late “looking” was critical for the successful completion of the shot. Two caveats apply to the approach of de Oliveira et al. (2008). First, they did not differentiate between fixations or saccades, which play a different role in vision. During fixations the gaze remains stable on a location within 1–3° of visual angle for a minimum of 100 ms allowing the brain to process the information being viewed, while during saccades vision is suppressed (Liversedge et al., 2006; Nystrom and Holmqvist, 2010; Marsman et al., 2012). de Oliveira collected data at 50 Hz, therefore each sample of “looking” had a duration of 20 ms, irrespective of being a fixation or a saccade. A second caveat surrounded how the location of “looking behavior” was determined. Looking behavior was coded using a 0 to 1 system, in which looking at the rim was given a score of 1, the net or the small square on the backboard 0.8, the backboard 0.6, all other locations 0.4, and no gaze behavior 0. In their final analysis, they defined looking using all the gaze with scores less than or equal to 6, thus the target encompassed a very large area that included not only the hoop, but the net and backboard as well.

### Location of Fixations

**FIGURE 1:**
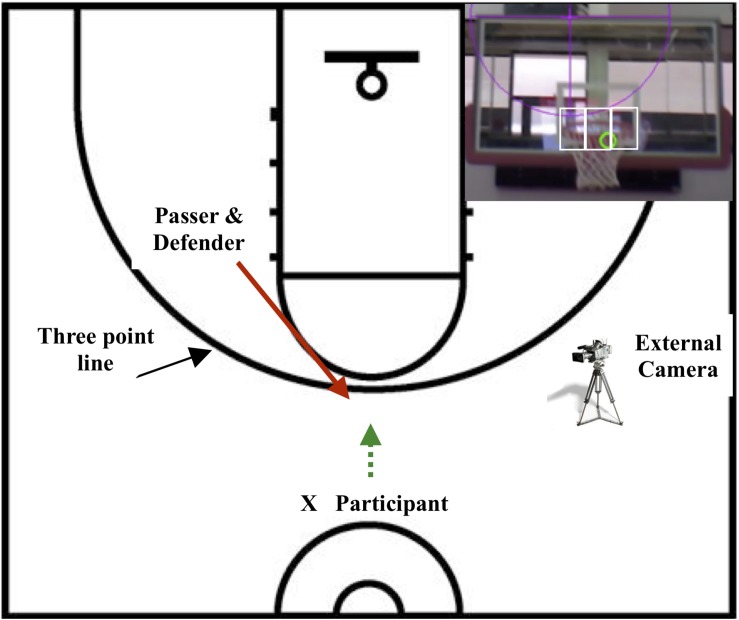
The experimental set-up showing the start position of the participant and the passer/defender relative to the three-point line. The hoop from the perspective of the participant is inset, showing the hoop left, hoop centre, and hoop right locations. The green circle shows the location of the gaze cursor subtending the target by 1.25° of visual angle from a distance of 23 ft (7.01 meters).

Studies have varied in how they have detected and analyzed the location of fixation during the basketball shot, with no consensus about which location is most critical to success. Vickers (1996a, b) determined the location of fixations relative to seven areas (ball, hands, floor; front hoop, middle hoop, backboard, out of range (outside the backboard) and reported that the location of fixation had no relationship to where the ball eventually landed. In a QE training study, Harle and Vickers (2001) identified the QE relative to five locations (front rim, back rim, left rim, right rim, backboard) and found players increased the percent of QE on the back rim after QE training. de Oliveira (2016) defined looking as described above, while Klostermann et al. (2017, p. 3) defined the QE as the last fixation anchored “for at least 100 ms at the basketball hoop”. We determined the location of the QE on seven locations: the ball, passer, backboard, net and the hoop divided into three locations, centre hoop, left hoop, right hoop, with each section being 6″/15.24 cm wide (Figure 1). We divided the hoop into three areas as our goal was to determine if ego-centric control of the gaze was critical in achieving success. Perception of direction includes both allo-centric and ego-centric perception of space (Coren et al., 2004). Allo-centric vision encodes spatial information about objects relative to one another, for example the location of players on the court relative to one another, while ego-centric vision is defined as the perceived location of an object in space with respect to the observer as origin (Morgan, 1978). Three type of ego-centric perception have been identified, body-centric, head-centric, and gaze centric (Li et al., 2013). Although all three are important when performing a basketball shot, we concentrate on gaze-centric vision. When applied to the three-point shot, gaze-centric vision occurred when the QE was located on the centre of the hoop, versus non-centre locations. Since QE training studies that have emphasized focusing on the centre portion of the hoop have led to improved performance, we expected the ego-centric control of the gaze on the centre of the hoop would be a characteristic of higher performance (Harle and Vickers, 2001; Vine and Wilson, 2011).

### Effect of the Defender

National Basketball Association statistics show the average accuracy of the top 50 NBA players in the 2018 season in the undefended free throw was 92% compared to 37.8% for the defended three-point shot revealing the profound effect the defender has on accuracy (NBA, 2019b). Early eye tracking studies in the free throw and jump shot did not include a defender, while only a few studies have included a defender, leading to mixed results in terms of the defenders effect on accuracy and the duration of fixations. Gorman and Maloney (2016) and Klostermann et al. (2017) found the defender reduced accuracy, while van Maarseveen et al. (2018) found the defender was not a significant factor. Klostermann et al. (2017) also found no differences in the QE duration during the undefended condition, but a longer QE duration in the defended condition, while van Maarseveen et al. (2018) found players who had the highest accuracy scores had a longer final fixation duration in the defended and undefended condition, while the lower scoring group had a longer duration only in the defended condition.

### Hypotheses

To date, the eye tracking literature in basketball and other motor tasks, does not suggest the number or percent of QE periods should differ by motor phase. We therefore hypothesized that there would be no significant difference in the number or percent of QE periods due to phase. Consistent with past QE studies, we expected the participants to have a longer QE duration during successful trials, and that greater success would occur during an early phase of the shot (QE catch, QE arm preparation, or QE arm flexion), rather than during a latter phase (QE arm extension, QE ball release). We also expected ego-centric control of the QE on the centre of the hoop to contribute to better performance than fixations on non-centre locations. Finally, we predicted the defender would have a negative impact on shooting accuracy, consistent with competitive results.

## Materials and Methods

### Participants

Twelve elite basketball players (8 male, aged 22.4 ± 2.2 years) were recruited with a combined average above 30% in the two and three-point during the previous season (Table 1). All played at the university or semi-professional level; eight were members of the team that won the Canadian university men’s championship the following season. The research protocol was approved prior to data collection by the Conjoint Ethics Committee of the University of Calgary, and all participants gave consent.

**TABLE 1:** Percent accuracy for each participant in the two and three-point jump shot in the previous season, and in the four tests (pre-test, undefended, defended, and post-test).

**Participants**	**Season 2 point %**	**Season 3 point %**	**Pre-test %**	**Undefended %**	**Defended %**	**Post-test %**	**4 Test Average %**
P1	29	23	50	50	40	48	47
P2	37	31	40	45	48	64	49
P3	39	27	50	45	52	34	45
P4	48	35	30	50	33	57	43
P5	53	43	30	48	48	52	45
P6	42	25	40	60	63	57	55
P7	59	36	60	83	52	64	65
P8	48	34	50	57	50	36	48
P9	43	36	70	80	48	78	69
P10	46	35	50	70	55	72	62
P11	44	33	50	52	48	57	52
P12	48	31	60	50	57	55	56
Average %	45	32	48	58	50	56	53

*The average for all participants is shown at the bottom.*

### Equipment

Gaze was recorded using an ASL Mobile Eye 5 eye tracker (Applied Sciences Laboratory, Bedford, MA, United States), and an external motor camera (Canon Vixia HF R42) that recorded the phases of the shooting action in the sagittal plane. The Mobile Eye is a light (76 g), glasses-mounted, monocular corneal reflection system that measures point of gaze with an accuracy and precision of 0.5° of visual angle. Both the gaze and motor videos were recorded at a rate of 30 Hz (33.33 ms/frame of video).

### Task and Protocol

Shots were taken from behind the three-point line on a regulation basketball court used in competition from a distance of 22–23 ft from the hoop (Figure 1). All shots were one-time shots, which occur when the player takes the shot immediately after receiving the pass without any attempt to dribble or take other evasive actions. One-time shots require precise timing and are among the most difficult and advanced shots in basketball. During the 2016 NBA season, approximately one half of the 402 three-point shots that Curry took were one time shots (FreeDawkins, 2019). Participants were instructed to step forward to receive the ball and shoot as quickly and accurately as possible from behind the three-point line. The pass was delivered by a highly skilled player/coach, who also acted as the defender. After a warm-up, a pre-test was performed without the eye tracker, followed by fitting the eye tracker and taking 3–5 practice trials until comfortable. Continuous shots were taken in counterbalanced conditions (undefended, defended) until 10 hits and 10 misses were recorded in each condition. A maximum of 40 shots were taken per condition and percent accuracy determined. During the defended condition, the defender actively challenged the participant, using an outstretched hand that was visible in the participant’s visual field during the defended trials. The post-test followed and was performed without the eye tracker. Total testing time was approximately 45 min. During data collection, the gaze and motor data were observed in real time on monitors to ensure calibration on each trial.

### Data Coding and Processing

The gaze and motor videos were synchronized using the Quiet Eye Solutions software (quieteyesolutions.com, 2010). A total of 430 trials were coded of the maximum 480 possible. Fifty trials were not included due to technical difficulties with the eye tracker and/or camera during data collection. An equal number of hits and misses were included for each participant per condition. The final data set consisted of 215 hits and 215 misses and 218 undefended and 212 defended trials. Trial onset (0 ms, 0%) was similar in each trial for the motor and gaze data. Trial onset occurred when the ball left the hand of the passer, and trial offset (100%) when the ball was released from the participant’s fingertips. The pass phase began with the first frame of video showing the ball leave the hands of the passer and ended with the frame prior to the ball first contacting the hands of the participant. The arm preparation phase began with the first frame showing the angle at the elbow increase [also called the dip (Penner, 2018), or loading the ball]. Arm flexion began when the angle at the elbow decreased as the ball was raised through the mid-line of the body and above the head. Arm extension began with the first frame showing the angle at the elbow increase until the ball left the finger-tips. Arm extension offset was similar to ball release, as beyond this point the participants had no control over the outcome of the shot. The occlusion phase began when the ball/hands/arm of the shooter entered the visual field and the target was no longer visible. The occlusion period ended when the target was visible.

Once the motor phases were entered into the Quiet Eye Solutions program, fixations and saccades were entered, in order, beginning at time 0. A fixation occurred when the participant’s gaze dwelled on a location for a minimum of 100 ms (3 frames of video) within 1.25° of visual angle (width of the cursor on the hoop shown in Figure 1). Each section of the hoop subtended a visual angle of 1.25° from a distance of 23 ft (7.01 meters) from the hoop as calculated by the Visual Angle Calculator available at Ellis (2009). The hoop was divided into three equal parts, each having an equal centre width of 6″ (15.24 cm). Within each third of the hoop, the athlete normally fixated the front, middle or back of the hoop. If the gaze cursor was located on an area between the three target areas, or on the edge of the rim, it was assigned to the area in which more than half the cursor was located (which was within the 0.5° of precision and accuracy of the eye tracker). For example, Figure 1 (inset) shows that more than half of the gaze cursor was located on the centre of the hoop, therefore it was coded as a fixation on hoop centre assuming three consecutive frames were located in the hoop centre area. If more than half the gaze cursor was located on the rim, then it was coded on the backboard or net.

A saccade occurred when the gaze moved rapidly between locations in two or more frames. Seven locations were coded: passer, ball, hoop centre, hoop right, hoop left, net, and backboard. Coding was carried out by two independent coders, and intra-class correlations were determined for the motor phases and QE onset, offset and duration. *R*-values ranged from 0.88 to 0.92.

### Isolating the Five QE Periods

The five QE periods were isolated using the Quiet Eye Solutions software, which has a function that detects the onset of the final fixation prior to the onset of a motor phase and automatically outputs the QE location, onset, offset, and duration. Each QE period was isolated separately, and then combined into a single data file. QE catch onset was the final fixation prior to the catch, and had an offset that occurred when the final fixation deviated off a location by more than 1.25° of visual angle or 3 frames (100 ms), a standard applied to all the QE offsets. QE arm preparation onset was the final fixation on a location prior to the angle at the elbow increasing. QE arm flexion onset began on a location prior to the angle at the elbow decreasing. QE arm extension onset was the final fixation on a location prior to the angle at the elbow increasing. QE ball release was initiated during arm extension prior to ball release. One limitation of the Quiet Eye Solutions software is that it duplicates a QE period when it extends across two or more motor phases. All duplicate QE periods were removed and the first was one retained, as it provided the most immediate visual guidance to the motor phase immediately following.

### Data Analysis

Data were analyzed and the results graphed using JMP 14.3 (JMP/SAS, 2019). Season and experimental accuracy were analyzed using ANOVA for test (pre-test, undefended, defended condition, post-test) and condition (undefended, defended). The number and percent of QE were analyzed by phase using nominal logistic regression. Motor phase onset, offset, duration, and QE phase onset, offset, and duration were analyzed in absolute (ms) and relative time (%) using a full-factorial repeated-measures linear mixed-effects ANOVA. Relative time was calculated by determining the percent (%) of a motor or QE variable as a function of total trial time. A mixed model ANOVA was most appropriate for the current study as it is a powerful method for handling missing observations and unbalanced designs, leading to more reliable conclusions, as well as accounting for repeated measures (Bagiella et al., 2000; Baayen et al., 2008). Fixed effects were condition (undefended, defended), outcome (hits, misses), location (hoop centre, non-centre), and participants (*n* = 12) were the random effect. Contrast of means was used to determine interaction effects. Effect sizes were calculated using partial η^2^ in accordance with Cohen’s *d*, with 0.10 considered a low effect, 30 a moderate effect, and 0.50 a large effect. The significance level was set at 0.05.

## Results

### 1.0 Percent Accuracy

Table 1 shows the percent accuracy for the two and three-point in the previous season, and for the pre-test, defended and undefended tests, and post-test. A significant difference was found for test, *F*_(3,33)_ = 3.04, *p* < 0.04, *d* = 0.22. Pre-test accuracy did not differ significantly in the undefended and defended conditions, and was lower confirming the eye tracker did not affect accuracy. Post-test accuracy did not differ from the undefended and defended conditions, confirming fatigue was not a factor. Undefended accuracy was higher (58%) than defended (50%), contrast of means, *F*_(1,33)_ = 4.55, *p* < 0.001.

### 2.0 Trial Duration, Motor Phase Onsets, Offsets and Durations and Occlusion

Trial duration was longer in the undefended than defended condition, *F*_(1,11.06)_ = 92.86, *p* < 0.0001, η_P_^2^ = 0.89, M undefended = 1417.96 ms (SE = 36.23 ms), M defended = 1270.65 ms (SE = 36.24). Table 2 presents the mean motor phase onsets, offsets and durations in absolute and relative time. No significant differences were found related to outcome by phase or condition. Significant differences were found for phase and also condition. Motor phase onsets differed in absolute time, *F*_(3,33.05)_ = 523.26, *p* < 0.0001, η_P_^2^ = 0.94, as did phase offsets, *F*_(3305)_ = 235.11, *p* < 0.0001, η_P_^2^ = 0.85 and durations, *F*_(3,33.03)_ = 18.01, *p* < 0.0001, η_P_^2^ = 0.37. Similar differences were found for relative time onset, *F*_(3,33.05)_ = 725.05, *p* < 0.0001, η_P_^2^ = 0.95, offset, *F*_(3,33.05)_ = 384.64, *p* < 0.0001, η_P_^2^ = 0.92, and duration, *F*_(3,33.05)_ = 19.45, *p* < 0.0001, η_P_^2^ = 0.37. The interaction of phase by condition was significant for onset in absolute time, *F*_(3,33.05)_ = 31.37, *p* < 0.0001, η_P_^2^ = 0.49, as well as offset, *F*_(3,33.05)_ = 14.21, *p* < 0.0001, η_P_^2^ = 0.31, and duration, *F*_(3,33.05)_ = 5.58, *p* < 0.003, η_P_^2^ = 0.15. Only phase duration differed in relative time, *F*_(3,33.05)_ = 11.02, *p* < 0.0001, η_P_^2^ = 0.25. The pass was delivered more slowly by the passer in the undefended than defended condition, *F*_(1,11.23)_ = 52.64, *p* < 0.0001, η_P_^2^ = 0.82, M undefended = 506.71 ms (SE = 7.93) and M defended = 438.97 ms (SE = 7.94). Shot release time included the arm preparation, arm flexion, and arm extension durations combined, and occurred earlier in the defended than undefended condition, *F*_(1,11)_ = 23.44, *p* < 0.02, η_P_^2^ = 0.005, and was slower in the undefended condition, M undefended = 860.92 (SE = 32.79 ms) and M defended = 780.94 (SE = 32.94 ms).

**TABLE 2:** Mean motor onsets, offsets and durations (ms, %) for the (1) the pass (as delivered by the passer), (2) arm preparation, (3) arm flexion, and (4) arm extension by condition.

		**Motor phases**							
		
		**(1) Pass**		**(2) Arm preparation**		**(3) Arm flexion**		**(4) Arm extension**	
					
		**Condition**		**Condition**		**Condition**		**Condition**	
					
		**Undefended**	**Defended**	**Undefended**	**Defended**	**Undefended**	**Defended**	**Undefended**	**Defended**
Motor Phases onset ms	Mean	0.00	0.00	539.27	473.26	901.21	795.44	1248.61	1108.78
	Standard Deviation	0.00	0.00	43.92	47.14	160.17	108.74	145.80	111.27
Motor Phases onset %	Mean	0.00	0.00	38.90	37.72	64.78	63.56	89.35	88.21
	Standard Deviation	0.00	0.00	3.87	3.04	10.23	8.64	3.84	4.58
Motor Phases offset ms	Mean	539.27	473.10	901.21	795.44	1248.61	1108.62	1396.93	1258.04
	Standard Deviation	43.92	47.11	160.17	108.74	145.80	111.58	157.03	122.46
Motor Phases offset %	Mean	38.90	37.71	64.78	63.56	89.38	88.19	99.99	100.00
	Standard Deviation	3.87	3.01	10.23	8.64	3.84	4.59	0.17	0.00
Motor Phases duration ms	Mean	505.81	439.61	361.92	322.15	347.39	313.17	148.26	149.16
	Standard Deviation	43.79	47.07	146.29	104.48	171.84	143.15	57.53	65.41
Motor Phases duration %	Mean	36.48	35.02	25.88	25.83	24.61	24.63	10.60	11.79
	Standard Deviation	3.69	2.94	9.34	8.24	11.03	10.16	3.84	4.58

**FIGURE 2:**
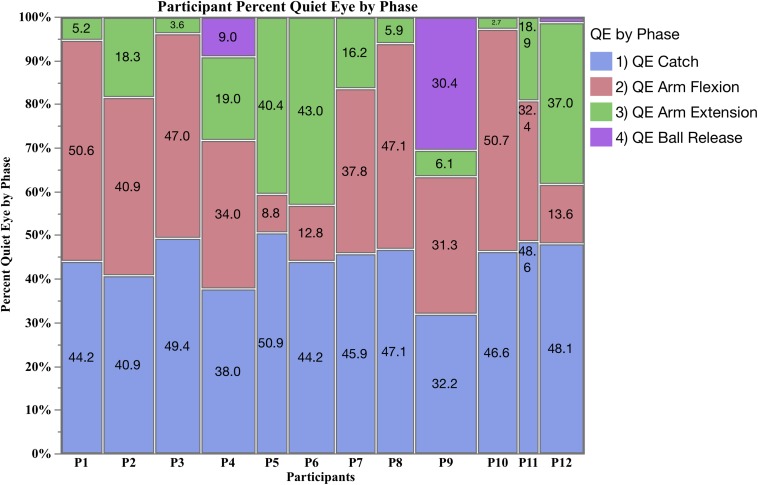
Mosaic plot of the percent of QE fixations for each participant by phase (QE catch, QE arm flexion, QE arm extension, and QE ball release).

Occlusion onset occurred earlier in the defended than undefended condition, *F*_(1,11)_ = 85.51, *p* = < 0.0001, *d* = 0.88, M defended = 1025.86 (SE = 44.06) and M undefended = 1174.60 (SE = 44.05). Occlusion offset was earlier in defended versus undefended condition, *F*_(1,11)_ = 9.93 *p* < 0.009, *d* = 0.52, M defended = 1372.57 (SE = 83.25) and M undefended = 1469.80 (SE = 83.23). Occlusion duration did not differ by condition, M undefended M = 294.30 ms (SE = 80.22) and defended M = 364.93 (SE = 81.80). Outcome was significantly affected by occlusion offset, *F*_(1,11)_ = 5.60 *p* < 0.04, *d* = 0.52. Hits occurred when occlusion offset was earlier, M hits = 1403.38 (SE = 82.42), and M misses, 1440.46 (SE = 82.42). The interaction of condition by outcome was not significant. In order to determine if occlusion may have played a role in initiating arm extension, arm extension onset was subtracted from occlusion onset. Occlusion onset occurred before arm extension in the undefended by 74.00 and 1.59 ms before in the defended, suggesting it may have played a role in initiating arm extension (however, see the results for QE duration Figure 2). A similar comparison for occlusion offset relative to ball release indicated that the target was occluded for 38.87 ms beyond ball release in the undefended condition, and 147.00 ms beyond in the defended condition.

### 3.0 Number and Percent of QE Fixations by Phase

A total of 944 QE fixations were found, which accounted for 87.08% of all fixations. Table 3 shows the percent of QE declined across the motor phases, with the highest percent occurring during QE catch, followed by QE arm flexion, QE arm extension, and finally QE ball release. There were no QE fixations during arm preparation, due to a rapid shift of gaze (saccade) to the target made by all participants immediately following QE catch offset. Our expectation that the proportion of QE fixations would be equal in each motor phase (25% per phase) was not upheld and differed significantly from the hypothesized values, χ^2^_(3,427.73)_ *p* < 0.0001. The one-way ANOVA was significant for the number of QE by phase, *F*_(3,47)_ = 19.91, *p* < 0.0001, η_P_^2^ = 0.75, indicating the target was increasingly difficult to fixate across the motor phases. Table 3 shows the percent of QE initiated in each phase that resulted in a hit or miss. The highest percent of hits were initiated during QE arm flexion (61%), followed by QE arm extension (31%), QE ball release (8.6%) and least for QE catch (0.003%).

**TABLE 3:** Number and percent of quiet eye fixations by motor phase, and percent of quiet eye initiated in a phase during hits and misses.

**QE fixation by phase**	**Number of QE fixations**	**Percentage of QE fixations**	**Percentage of QE on target hits**	**Percentage of QE on target misses**
QE Catch	412	43.6%	0.007%	0.003%
QE arm preparation	0	0%	0%	0%
QE arm flexion	322	34.1%	61%	60%
QE extension	165	17.5%	31%	31%
QE ball release	45	4.8%	7.9%	8.6%
Total	944 (87% of total fixations)	100%	100%	100%

### Individual Frequency of QE

Table 4 presents the mean frequency of QE per participant during the complete trial (four phases), and during the final three shooting phases. Frequency of QE averaged 2.22 per trial and did not differ due to condition, M undefended = 2.23 (SE = 0.03) and M defended = 2.20 (SE = 0.03), or outcome, M hits = 2.23 (SE = 0.03), and M misses = 2.20 (SE = 0.03). QE frequency averaged 1.25 fixations during the final three phases and did not differ by condition, M undefended = 1.26 (SE = 0.03), M defended = 1.23 (SE = 0.03), or outcome, M hits = 1.26 (SE = 0.03), M misses = 1.24 (SE = 0.03). One participant (A9) had a higher frequency of QE fixations, averaging 3.11 over all phases and 2.11 as the shot was taken. Overall, the results show that 11/12 participants’ averaged one opportunity to fixate the target after the pass was received.

**TABLE 4:** Mean frequency of QE during all phases, and during the final three shooting phases, by participant (P1–P12).

	**QE frequency (All phases)**	**QE frequency (Final 3 phases)**
		
**Participants**	**Mean**	**Mean**
P1	2.08	1.10
P2	2.45	1.45
P3	2.02	1.05
P4	2.53	1.53
P5	1.97	1.00
P6	2.26	1.26
P7	2.14	1.20
P8	2.00	1.06
P9	3.11	2.11
P10	2.00	1.03
P11	1.85	1.05
P12	2.03	1.08
All	2.22	1.25

### Percent of QE by Phase by Participant

The mosaic plot in Figure 2 shows the percent of QE initiated by each participant during QE catch, QE arm flexion, QE arm extension and QE ball release. During QE catch, all participants had a consistently high percentage, ranging from 35.2 to 50.9% of the total QE. During the final three phases, participants initiated a QE during the arm flexion or extension phase, but given the mean QE frequency was 1.25, there was not enough time for a fixation to occur in both phases in one trial. Nine participants (P1, P2, P3, P4 P7, P8, P9, P10, and P11) primarily fixated the target during arm flexion (red), initiating a minimum 31.3% of their total QE during this phase, and a low percent of QE during arm extension (green). Three participants (P5, P6, and P12) had a later QE during arm extension, minimum 37%. Three participants (P4, P9, and P12) initiated a QE during all three shooting phases, and were the only athletes to initiate a QE prior to ball release (purple). P9 was unique in consistently initiating a QE prior to ball release, accounting for 35 of the total 45 QE periods observed. Due to the low number of fixations during QE ball release, a formal analysis could not be carried out, but a descriptive analysis is provided after the ANOVA results for QE catch, QE arm flexion and QE arm extension are presented.

### 5.0 QE Location

**FIGURE 3:**
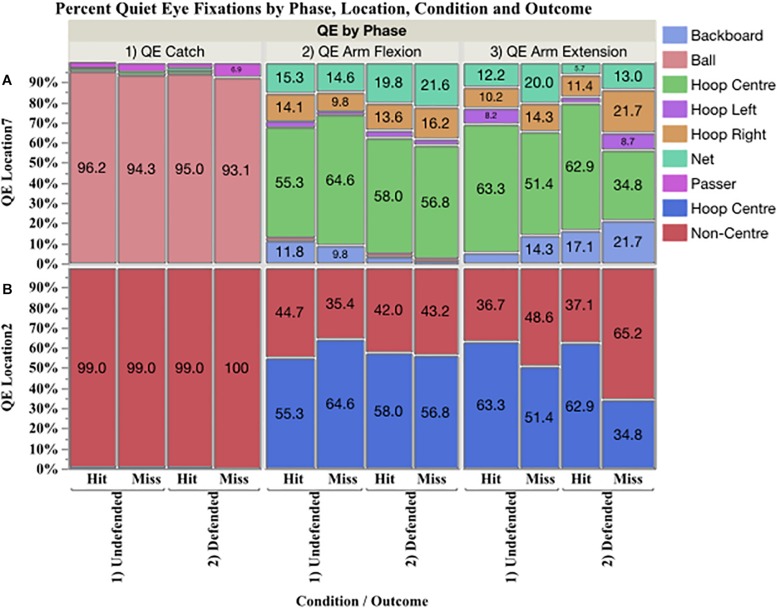
Mosaic plot showing in the top panel **(A)** the percent of QE fixations to seven locations (QE location 7: backboard, ball, hoop centre, hoop left, hoop right, net, and passer), and in the bottom panel **(B)** to two locations (QE location 2: hoop centre and non-centre) by phase, condition and outcome.

The mosaic plot shown in Figure 3 shows the percent of QE fixations by phase on seven locations in the top panel (A), and two locations (hoop centre, non-centre) in the bottom (B) by condition and outcome. During QE catch, the ball accounted for 93.1–96.2% of the total. Fixations were also found on the passer and the hoop, but these accounted for a low percent of the data. During QE arm flexion and QE arm extension, the primary locations fixated, in order, were hoop centre, net, backboard, hoop right, and hoop left.

**FIGURE 4:**
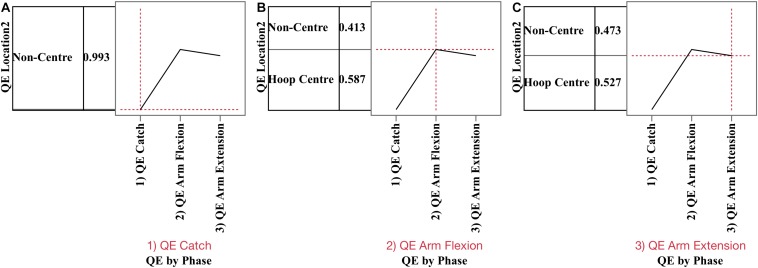
Prediction profiles for phase by location (hoop centre, non-centre) for QE catch **(A)**, QE arm flexion **(B)**, and QE arm extension **(C)**. During (1) QE catch, the probability of not fixating the target was 0.993, due to virtually all fixations being on the ball prior to the catch. During (2) QE arm flexion the probability of the QE being on hoop centre was 0.589 compared to 0.413 for non-hoop locations. During (3) QE arm extension the probability of fixating the hoop centre was 0.527 and non-centric location 0.473.

Percent of QE on hoop centre and non-centre locations (Figure 3B) were analyzed using nominal logistic regression. The probability of fixating the hoop centre versus non-centre locations was affected by phase, χ^2^_(3,427.73)_ *p* < 0.0001, but not by outcome or condition. Figure 4 presents the prediction profile for location by phase. During QE catch (A), the probability of fixating non-hoop locations was 0.99 and these fixations were primarily on the ball. During QE arm flexion (B) the probability of fixating the hoop centre was 0.587 and 0.413 for non-centre locations, while during QE arm extension (C), the probabilities declined to 0.527 and 0.473, respectively.

### QE Onset, Offset and Duration by Phase

Table 5 presents the mean QE onset, offset and duration by phase and condition in both absolute (ms) and relative (%) time. QE catch was analyzed separately from QE arm flexion and QE arm extension, due to its different functions performed, i.e., to catch the ball versus taking the shot. The QE catch data were analyzed using a repeated mixed-effects ANOVA by condition, location and outcome.

**TABLE 5:** Mean QE onset, offset and duration (with standard error) by phase and condition in both absolute (ms) and relative time (%).

		**Quiet Eye by Phase**					
		
		**(1) QE catch**		**(2) QE arm flexion**		**(3) QE arm extension**	
				
		**Condition**		**Condition**		**Condition**	
				
		**Undefended**	**Defended**	**Undefended**	**Defended**	**Undefended**	**Defended**
QE onset (ms)	Mean	31.46	26.92	739.77	656.47	936.12	855.96
	Standard Error	5.24	4.10	10.18	8.11	20.04	16.66
QE offset (ms)	Mean	438.76	357.53	1050.90	893.08	1103.17	993.01
	Standard Error	4.99	5.87	14.38	11.67	18.27	14.87
QE duration (ms)	Mean	407.13	330.54	311.10	237.02	168.58	136.98
	Standard Error	5.65	5.57	13.72	9.47	12.12	6.73
QE onset %	Mean	2.25	2.17	53.11	52.35	66.95	67.35
	Standard Error	0.38	0.33	0.72	0.59	1.14	1.07
QE offset %	Mean	31.63	28.39	74.68	70.84	78.80	78.12
	Standard Error	0.39	0.45	0.60	0.56	0.82	0.86
QE duration %	Mean	29.09	25.58	21.57	18.49	11.97	10.77
	Standard Error	0.47	0.49	0.78	0.62	0.78	0.49

#### QE Catch

**FIGURE 5:**
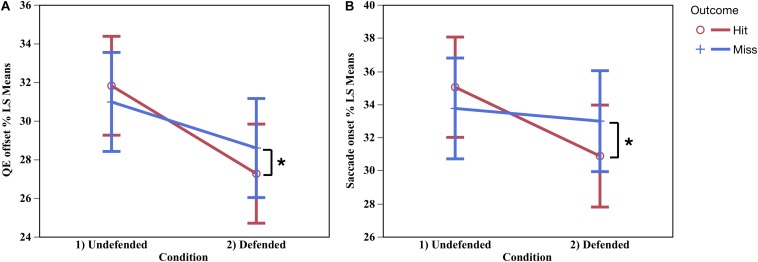
QE catch offset occurred earlier during hits in the defended condition, and later in the undefended condition **(A)**. Hits occurred in the defended condition when there was an early saccade onset to the target, while in the undefended condition saccade onset occurred later **(B).** ^∗^Significant difference *p* < 0.05.

No differences were found for QE onset, but QE offset differed by condition by outcome in relative time, *F*_(1,10.23)_ = 5.06, *p* < 0.002, η_P_^2^ = 0.33, contrast of means, *F*_(1,29.29)_ = 18.35, *p* < 0.05, and neared significance in absolute time, *F*_(1,10.72)_ = 3.71, *p* < 0.08. Figure 5A shows that during hits QE offset (%) occurred earlier in the defended than the undefended condition. QE catch duration (%) was shorter in the defended than undefended condition, in both relative *F*_(1,10.89)_ = 10.89, *p* < 0.006, η_P_^2^ = 0.52, and absolute (ms) time, *F*_(1,11.01)_ = 59.31, *p* < 0.0001, η_P_^2^ = 0.85. During defended hits, participants ceased tracking on the ball an average of 109 ms before the catch in the undefended condition, and 89 ms in the defended.

#### Saccade to the Target

Immediately following QE catch offset, a saccade was made to the target. Saccade onset differed by condition and outcome in both absolute *F*_(1,8.48)_ = 4.51, *p* < 0.03, *d* = 0.34, and relative time, *F*_(1,8.48)_ = 5.71, *p* < 0.04, *d* = 0.40. Figure 5B show the saccade onset occurred earlier during hits in the defended condition than undefended. Saccade duration was longer in the defended than undefended condition, in both absolute, *F*_(1,7.43)_ = 9.61, *p* < 0.02, *d* = 0.56, and relative time, *F*_(1,8.90)_ = 18.01, *p* < 0.002, *d* = 0.67, M undefended = 195.66 ms (SE = 23.39), M defended = 231.28 ms (SE = 23.98).

### QE Arm Flexion and QE Arm Extension

Since a goal of the study was to determine which QE period was most effective, the QE arm flexion and QE arm extension data were analyzed using a repeated mixed-effects ANOVA by QE phase, condition, location and outcome.

#### QE Onset

**FIGURE 6:**
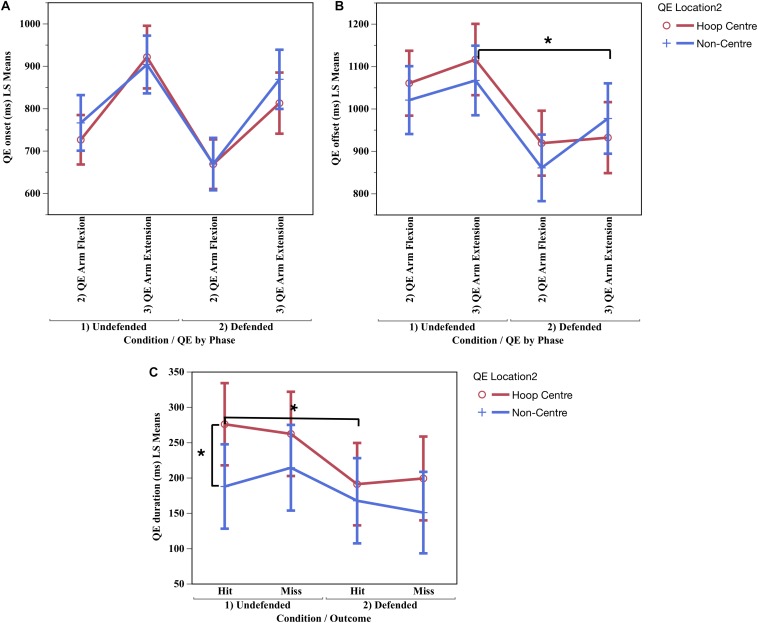
Mean quiet eye onset **(A)** and quiet eye offset **(B)** by condition, phase and location, and quiet eye duration **(C)** by condition, location and outcome. ^∗^Significant difference *p* < 0.05.

Significant main effects were found for QE phase onset in absolute, *F*_(__1,10.57)_ = 31.46, *p* < 0.0002, η_P_^2^ = 0.75, and relative time, *F*_(1,10.86)_ = 40.19, *p* < 0.0001, η_P_^2^ = 0.79. Then main effect for condition differed in absolute time, *F*_(1,12.02)_ = 20.86, *p* < 0.0006, η_P_^2^ = 0.63; and for the interaction of condition x phase x location in both absolute, *F*_(1,168.4)_ = 4.08, *p* < 0.04, η_P_^2^ = 0.03, and relative time, *F*_(1,219.4)_ = 4.54, *p* < 0.03, η_P_^2^ = 0.02, Figure 6A shows the participants initiated a fixation earlier during QE arm flexion than QE arm extension in both the undefended and defended conditions on hoop centre and non-centre locations.

#### QE Offset

A significant difference was found for QE phase in absolute, *F*_(1,10.45)_ = 34.57, *p* < 0.0001, η_P_^2^ = 0.76, and relative time, *F*_(1, 10)_ = 11.37, *p* < 0.008, η_P_^2^ = 0.53. The interaction of QE phase × condition × location was significant in absolute time (ms), *F*_(1,144.3)_ = 5.19, *p* < 0.02, η_P_^2^ = 0.03, and relative time, *F*_(1,147.3)_ = 7.22, *p* < 0.006, η_P_^2^ = 0.05. Figure 6B shows the QE offset was maintained later on the hoop centre during QE arm flexion and QE arm extension in the undefended condition, but occurred significantly earlier in both phases in the defended condition. The earlier QE offsets during the defended condition could have been caused by ball occlusion, or by pressure from the defender. Since ball occlusion also occurred in the undefended condition, then the defender was the most likely cause for the early termination of fixations on hoop centre and non-centre locations. The QE offsets in both conditions occurred well before ball release, which occurred at 1430.28 ms in the undefended condition and 1291.37 ms in the defended (Table 2).

#### QE Duration

A significant difference was found for QE phase in absolute, *F*_(1,11.52)_ = 36.79, *p* < 0.0001, η_P_^2^ = 0.76, and relative time, *F*_(1,10.34)_ = 48.76, *p* < 0.0001, η_P_^2^ = 0.80. The interaction of condition × location × outcome was significant in absolute time, *F*_(1,58.09)_ = 4.37, *p* < 0.04, η_P_^2^ = 0.08, and neared significance for relative time, *F*_(1,173.50)_ = 3.22, *p* < 0.07. Figure 6C shows the QE duration was longer during hits on hoop centre (M = 275.53 ms) than on non-centre locations (M = 187.39 ms) in the undefended condition (contrast of means, *F*_(1,28.40)_ = 12.21, *p* < 0.03), but that a shorter duration QE occurred during hits in the defended condition (M = 190.72 ms). The shorter QE durations in the defended condition could have been due to occlusion, or pressure from the defender. Since occlusion occurred in both conditions, the shorter QE duration was most likely caused by pressure from the defender.

### QE Ball Release

Participant P9 was unique in using three QE periods per trial, one during the pass, one during arm flexion and one prior to ball release (Figure 2). In both the defended and undefended condition his trial duration was longer than the other participants, 1805.92 ms in the undefended condition, and 1546.78 in the defended, compared to 1430.26 and 1291.37 ms, respectively, for the other participants. Review of his video data showed his gaze deviated off the hoop centre to the left hoop after QE arm flexion, followed by a saccade back to hoop centre, and a final QE on hoop centre prior to ball release. P9 had the highest accuracy (80%) during the undefended condition, but was 6th overall at 48% during the defended condition (Table 1). Given he consistently fixated the target prior to ball release, then he would be classified as a high-style shooter as defined by Oudejans et al. (2002).

## Discussion

Our goal in this study was to investigate three areas of uncertainly about the role of vision in basketball shooting, specifically the timing of QE fixations, their location, and the role of the defender. We also sought to overcome a criticism of past QE studies, which have reported only one QE period. We sought to alleviate this problem by analyzing five QE periods, with each initiated before a biomechanical phase of the one-time basketball shot: QE catch, QE arm preparation, QE arm flexion, QE arm extension, and QE ball release. At the outset, we expected a longer QE duration during successful trials, which would be initiated during the early phases of the shot rather than during the latter phases. We also expected that ego-centric control of the QE on the centre of the hoop would contribute to better performance than fixations on non-centre locations, and that the defender would have a negative impact on shooting performance. We did not expect a significant difference in the number or percent of QE fixation in each phase. For the most part our expectations were upheld, but with some notable exceptions discussed below.

### Percent Accuracy

Percent accuracy was lower in the defended condition than undefended, 50% compared to 58%, which agrees with competitive statistics, and also studies by Gorman and Maloney (2016) and Klostermann et al. (2017)

#### Effect of the Defender

The defender not only negatively affected accuracy, but also the duration of the motor phases and QE periods. The duration of the arm preparation, flexion, and extension motor phases were lower in the defended versus the undefended condition (Table 2), as were the QE durations, whether calculated in absolute or relative time (Table 5). Release time was also lower in the defended condition (780.94 ms) than in the undefended (860.92). These results agree with those reported by Waters (2017), who determined the release time of five top NBA shooters from catch to ball release ranged from a low of 770 ms to a high of 820 ms.

### Percent of QE Across the Motor Phases

A total of 944 QE fixations were found, which accounted for 87.1% of total fixations. At the outset we expected to find no significant differences in the percent of QE fixations by phase. Contrary to our expectation, we found the percent of QE fixations differed significantly across the phases, with the highest percent/number occurring during QE catch (43.6%; 412 QE fixations out of 430 trials), followed by QE arm flexion (34.1%, 322 QE fixations), QE arm extension (17.5%, 165 QE fixations) and least for QE ball release (4.8%, 45 QE fixations). We found that no QE fixations were initiated prior to the arm preparation phase due to a saccade made by the participants to the target immediately after tracking on the ball ceased. The exceptionally low number of fixations during QE ball release was unexpected, and showed that the three-point shot is taken under such extreme time and defensive pressure that sustaining a fixation until the ball is released is very difficult.

### Participant Frequency of QE

Frequency of QE averaged 2.22 per trial, and 1.25 per participant during the final three shooting phases, meaning most participants had only one opportunity to fixate the target after the ball was caught. Percent of QE initiated by each participant by phase, showed that nine participants initiated their QE primarily during arm flexion, and three primarily during arm extension (Figure 2). Three participants were able to initiate a QE prior to ball release, and most of these were taken by one participant (P9), who was unique in consistently initiating a QE prior to ball release. A review of P9’s gaze videos showed that he used three QE periods, the first during QE catch, the second during QE arm flexion, and the final during QE ball release. He had a very long duration QE on hoop centre during arm flexion, but his gaze drifted to the left hoop during arm extension, followed by re-fixating the hoop centre prior to ball release. Since it takes time to re-fixate a location, this increased his trial duration to an average of 1803.92 ms in the undefended condition and 1546.78 ms in the defended, compared to 1430.26 and 1291.37 ms, respectively, for the other participants (Table 2). During the undefended trials he had exceptional accuracy (80%, 1st), but during the undefended condition his accuracy fell to 48%, 6th overall. In many respects, P9 exhibited the visual behavior described by Oudejans et al. (2002) and de Oliveira (2016) for high style shooters, as he did maintain fixation on the target through to the release of the ball, during both the defended and undefended trials. Overall, P9 shot was slower in delivering the shot than the other participants, his release time averaged 1023.10 ms in the defended condition, compared to 756.72 ms for the other participants.

For 11/12 participants, it is important to consider the offset of their QE arm flexion and QE arm extension (Table 5) relative to ball release (Table 2 arm extension offset). In the defended condition, QE arm flexion offset occurred at 893.08 ms, or 70.80% of trial time, and QE arm extension offset at 993.01 ms, or 78.12% of trial time (Table 5). Since ball release occurred at 1291.37 ms in the defended condition, this meant the target was not visible for 398.29 ms if QE arm flexion was the final fixation, and 298.36 ms if QE arm extension was used. These results also show that, except for P9, most participants used a low style of shooting rather than the high style as described by Oudejans et al. (2002).

### Effect of QE Location

During QE catch, participants primarily fixated the ball, and had very few fixations on the passer or the hoop. They also immediately made a saccade to the hoop thus lying to rest the idea that players can see the target during the pass, since during a saccade vision is suppressed. They also did not track the ball to their hands, but ceased tracking 109 ms before the catch in the undefended condition, and 89 ms in the defended. Critically, a long duration QE during hits occurred when the final fixation was located on the centre of the hoop, versus non-centre locations. The probability of being able to fixate the centre of the hoop was highest during QE arm flexion, rather than during QE arm extension or QE ball release (Figure 4). A weight of evidence therefore shows that ego-centric control of the QE during arm flexion was a factor in accuracy in the three-point shot as 61% of hits were initiated on the target during arm flexion, compared to 0.007% during the catch, 31% during arm extension, and 7.70% during ball release.

Why was a long duration ego-centric QE during arm flexion a factor in performance? Nakashima et al. (2015, p. 2) defines ego-centric spatial perception as “the perception of direction or position of oneself based on visual information acquired in the visual field.” Results showed that the participant’s focus was entirely on the ball prior to the catch, followed by a saccade during which vision was suppressed. Therefore vision of the target did not become possible until the onset of QE arm flexion, which occurred half way through the trial between 650 and 700 ms, or 50% of trial time. Following this, on-going feedback of the target did not occur for 11 of 12 participants during the final 300 ms before ball release. A weight of evidence therefore suggests the three-point shot is under automatic motor program control, in which a highly developed neural network is activated and the movement carried out without on-going visual feedback of the target during arm extension and ball release. The brief duration of the extension motor phase, which averaged 148–149 ms, and therefore qualifies as a ballistic movement and adds evidence in support of open loop control and motor program control (Table 2). We also must conclude that since all the participants ceased tracking the ball before the catch, and 11/12 ceased fixating the target during the final 300 ms of the shooting action, then optic flow and tau, as defined by Lee (1976, 2009), does not explain how accurate shots were made in the three point shot.

Since 11/12 participants did not fixate the target during the final 300 ms of the shooting action, then how was accuracy maintained during this time. Research shows that long-term memory of spatial locations persists when ego-centric vision is used to locate targets in space. Henriques et al. (1998) and Schutz et al. (2013) found that ego-centric target representation persisted in long term memory for up to 12 s when a reach movement to delayed targets was made. They concluded that “ego-centric target representations can persist for at least several seconds instead of becoming unavailable immediately after the target vanishes” Schutz et al. (2013, p. 46). Their results may also explain why ego-centric vision was more successful than non-centric vision. The QE non-centric fixations were much briefer than fixations on hoop centre, and were also to more locations. This meant there was less time to encode the location of the target, leading to non-optimal target commands to the shooting arm and hands as the ball was released.

### How Do the Five QE Periods Add to Our Existing Knowledge?

Given that five QE periods were isolated, it is interesting to speculate a few ways they add to our existing knowledge about the technical and strategic requirements needed to perform the three point shot effectively. What does knowing about the timing of the QE periods, their onset, offset, and location during successful and unsuccessful trials add to the game and future research? During QE catch, all the athletes fixated the ball, followed by a saccade to the hoop before the ball was caught, on average 109 ms before the catch in the undefended condition, and 89 ms in the defended. We can therefore speculate, that consistent with coaching (Wissel, 2011) and research (Ripoll et al., 1986; Marques et al., 2018) that the function of QE catch was to prepare the hands to catch the ball as early as possible, followed by an early offset of E catch and a rapid shift in gaze (saccade) to the hoop. But consider the effect of an alternate gaze strategy? What would have happened if the participants had fixated the target during the early pass, and then fixated the ball up to the moment it was caught, a strategy often recommended by coaches (USA Basketball, 2010). In the current study, the participants did not look the ball into their hands, but instead gained approximately 100 ms by shifting their QE to the hoop early before the catch, results that were directly related to accuracy. During QE arm flexion, the participants fixated the hoop for the first time using a long duration QE fixation that was initiated during the latter part of the arm preparation phase. Since this occurred relatively late in each trial this meant the location of the hoop had to be stored in memory for half a second or more. It therefore may be advisable to teach athletes to visually locate the hoop before the pass begins. This requires the athlete develop the decision-making and footwork skills to move into position and acquire information about the target before the pass begins. Gaze that occurs prior to critical events in basketball and other sports is an area that is receiving increased research attention (Okazaki et al., 2015, p. 12). Vater et al. (2019) provide a meta-analysis of the role and importance of peripheral vision across various sports, while in basketball specifically van Maarseveen et al. (2018), p. 250) found that “peripheral vision may serve a significant role in decision making *in situ*, whereas players mainly relied on central vision to execute an action.” The results of the current study and that of other eye tracking studies show the sequence of gaze matters in daily life (Henderson, 2003, 2017) and in QE training studies in sport and medicine (Causer et al., 2014a, b; Miles et al., 2015a, b; Lebeau et al., 2016). Elite athletes have found the best way to time the onset of their QE fixations and saccadic movements to optimally acquire task information at critical times during the movement. Lower level performers benefit from knowing about the experts’ sequence of gaze and optimal QE timing. Finally, from the defensive perspective, strategies that disrupt specific QE periods may reduce the effectiveness of the three-point shooter. Defensive strategies should include disruptive defense prior to and during QE catch, and defensive pressure in the visual field of the shooter during QE arm flexion and QE arm extension (Okazaki et al., 2015, p. 12–13; Rojas et al., 2000).

### QE Training Program

Figure 6C shows the QE arm extension duration in the defended condition during hits was very brief (average 190.72). Since this may lead coaches and athletes to attempt to develop a short duration QE in practice, we wondered if participants who had a low QE duration during the undefended condition had a higher percentage during the past season than those with a longer QE duration. We created two sub-groups (Low QE and High QE) based on their quiet eye duration in the undefended condition. Those classified as Low QE had a quiet eye duration ≤250 ms, and the High QE eye group >250 ms. Participants in the High QE group (*n* = 6) had a higher three-point average the previous season, average 35% than those in the Low QE group (*n* = 6) average 31%, suggesting the ability to focus for a longer duration during undefended practice conditions leads to better performance under the extreme pressure of competition. Athletes who develop a long duration QE in practice may have developed a neural network that is more easily adapted to handle high pressure conditions, but the opposite may not apply when the only QE duration an athlete possesses is very brief. Athletes who develop a low duration QE in practice may be less able to increase or decrease the quiet eye duration in response to the variable conditions of competition. Based on the results of this study, a QE training program in the basketball one-time three-point shot is recommended as follows:

(1) **For the passer:** The pass is critical to success in the three-point shot. The ball should be aimed at the shooter’s hand, or a location that he or she prefers. For right hand shooters, the pass should come from the left side of the court (facing the hoop), as this allows the pass to be caught on the shooting hand, and for left hand-shooters vice versa.
(2) **For the shooter:** Keep your eyes on the ball as it leaves the passer’s hand and track it closely.
(3) As early as possible, shift your gaze rapidly to the centre of the hoop and no other location.
(4) Maintain a fixation on the centre of the hoop for a half second as the shot is prepared and released. Focus on the centre front, centre middle or the centre back hoop - but only on one of these locations per shot.
(5) As you shoot, the ball and your shooting hand and arm should come up through the mid-line of your body and occlude (hide) the hoop as you shoot.
(6) Shoot as quickly and fluidly as possible.

## Limitations and Recommendations

The main limitation in the current study is the low number of participants with three-point shooting averages high enough over a full season to be classified as experts (*N* = 12). This is a common problem in expertise research, where the number of experts is usually relatively low. We expect this problem to improve in the coming years as more athletes perfect the three-point shot. A second limitation is that the results apply to the three-point shot, but we encourage studies in other basketball shots, as well as other motor tasks in which multiple quiet eye periods can be isolated, each prior to a biomechanical phase of the movement. It is critical an equal number of successful and unsuccessful trials be included, while leaving free to vary other conditions, such as the timing of the quiet eye periods, and the location of the quiet eye in each phase, as was done in current study. We realize this is a new experimental paradigm, but one we feel it will open up new avenues of understanding not only the nature of motor expertise, but also motor learning and control, and the importance of empirically defining the moment of optimal focus and attention.

## Conclusion

The results of this study greatly expand the explanatory power of multiple QE periods, with each initiated prior to the onset of a specific phase of the movement. Not only is perception-action coupling investigated relative to more biomechanical phases, but other factors are considered across the entire trial, such as the timing of QE fixations across the motor phases, the location of the QE in each phase, and the onset, offset and duration of each QE period by phase. To our knowledge this is the first study to show that the ability to fixate a target declines with the phases of the movement when extreme pressure is encountered. This is also the first eye tracking study to show that aiming to a far target is aided when ego-centric gaze control of the QE occurs prior to a specific phase of the movement. We also show that in the three-point shot there is an optimal moment when a fixation on the hoop centre had its greatest impact, and this occurred during QE arm flexion. We can therefore speculate that an elite athlete like Stephen Curry tracks the ball closely as it leaves the passers hand, followed by an early QE catch offset, an early saccade to the target, and an early QE fixation on the centre of the hoop before and during arm flexion for a duration of around 300 ms, and a rapid, automatic shooting action that is oblivious to the actions of the defender.

## Data Availability Statement

The datasets generated for this study are available on request to the corresponding author.

## Ethics Statement

The studies involving human participants were reviewed and approved by the research protocol was approved prior to data collection by the Conjoint Ethics Committee of the University of Calgary, and all participants gave consent. The patients/participants provided their written informed consent to participate in this study.

## Author Contributions

JV, JC, and DV made substantial contributions to conception and design, acquisition of data, and interpretation of data. JV carried out the analysis and drafted the manuscript. JC and DV participated in revising it critically for important content and final approval of the version submitted. All authors accepted the final manuscript.

## Conflict of Interest

The authors declare that the research was conducted in the absence of any commercial or financial relationships that could be construed as a potential conflict of interest. The reviewer SR declared a past collaboration with one of the authors, JV, to the handling Editor.
